# Global Variations in the Mineral Content of Bottled Still and Sparkling Water and a Description of the Possible Impact on Nephrological and Urological Diseases

**DOI:** 10.3390/jcm10132807

**Published:** 2021-06-27

**Authors:** Simone J. M. Stoots, Guido M. Kamphuis, Rob Geraghty, Liffert Vogt, Michaël M. E. L. Henderickx, B. M. Zeeshan Hameed, Sufyan Ibrahim, Amelia Pietropaolo, Enakshee Jamnadass, Sahar M. Aljumaiah, Saeed B. Hamri, Eugenio Ventimiglia, Olivier Traxer, Vineet Gauhar, Etienne X. Keller, Vincent De Coninck, Otas Durutovic, Nariman K. Gadzhiev, Laurian B. Dragos, Tarik Emre Sener, Nick Rukin, Michele Talso, Panagiotis Kallidonis, Esteban Emiliani, Ewa Bres-Niewada, Kymora B. Scotland, Naeem Bhojani, Athanasios Vagionis, Angela Piccirilli, Bhaskar K. Somani

**Affiliations:** 1 Department of Urology, Amsterdam UMC, AMC, University of Amsterdam, 1105 Amsterdam, The Netherlands; g.m.kamphuis@amsterdamumc.nl (G.M.K.); m.m.henderickx@amsterdamumc.nl (M.M.E.L.H.); 2 Department of Urology, Freeman Hospital, Newcastle NE7 7DN, UK; robgeraghty@btinternet.com; 3 Department of Internal Medicine, Section Nephrology, Amsterdam UMC, AMC, University of Amsterdam, 1105 Amsterdam, The Netherlands; l.vogt@amsterdamumc.nl; 4 Department of Urology, Kasturba Medical College and Hospital, Manipal Academy of Higher Education, Manipal, Karnataka 576104, India; zeeshanhameedbm@gmail.com (B.M.Z.H.); sufyan.ibrahim2@gmail.com (S.I.); 5 Department of Urology, University Hospital Southampton NHS Trust, Southampton SO16 6YD, UK; amelia.pietropaolo@uhs.nhs.uk (A.P.); enakshee@gmail.com (E.J.); bhaskarsomani@yahoo.com (B.K.S.); 6 Department of Urology, Ministry of the National Guard—Health Affairs, Riyadh 11426, Saudi Arabia; jumaiahsa@gmail.com (S.M.A.); sbinhamri@gmail.com (S.B.H.); 7 Division of Experimental Oncology/Unit of Urology, IRCCS Ospedale, Urological Research Institute, San Raffaele, 20132 Milan, Italy; eugenio.ventimiglia@gmail.com; 8 Department of Urology, Sorbonne University, GRC #20 urolithiasis, 75006 Paris, France; traxer.olivier@gmail.com; 9 Department of Urology, Ng Teng Fong General Hospital, Singapore 609606, Singapore; vineetgaauhaar@gmail.com; 10 Department of Urology, University Hospital Zurich, University of Zurich, 8091 Zurich, Switzerland; etienne.xavier.keller@gmail.com; 11 Department of Urology, AZ Klina, 2930 Brasschaat, Belgium; vincent.de.coninck@klina.be; 12 Department of Urology, University Clinical Center of Serbia, University of Belgrade, 11000 Belgrade, Serbia; odurutovic@gmail.com; 13 Department of Urology, Saint-Petersburg State University Hospital, 197022 Saint Petersburg, Russia; nariman.gadjiev@gmail.com; 14 Department of Urology, Addenbrooke’s Hospital, Hills Road, Cambridge CB2 0QQ, UK; lauriandragos@yahoo.com; 15 Department of Urology, Marmara University Hospital, Marmara University School of Medicine, Istanbul 34854, Turkey; dr.emresener@gmail.com; 16 Department of Urology, Redcliff Hospital, Brisbane QLD 4012, Australia; nickrukin@hotmail.com; 17 Department of Urology, ASST Fatebenefratelli-Sacco, Luigi Sacco University Hospital, 20157 Milan, Italy; michele.talso@gmail.com; 18 Department of Urology, University of Patras, 26504 Patras, Greece; pkallidonis@yahoo.com (P.K.); thanos_vagionis@hotmail.gr (A.V.); 19 Department of Urology, Fundació Puigvert, Autonomous University of Barcelona, 08025 Barcelona, Spain; emiliani@gmail.com (E.E.); angelapiccirilli@yahoo.it (A.P.); 20 Department of Urology, Medical University of Warsaw, 02-091 Warsaw, Poland; ewa.bres@gmail.com; 21 Department of Urology, University of California Los Angeles (UCLA), Los Angeles, CA 90095, USA; kscotland@mednet.ucla.edu; 22 Department of Urology, University of Montreal, Montreal, QC H2X 0A9, Canada; naeem.bhojani@gmail.com

**Keywords:** kidney stone disease, mineral water, mineral composition, drinking water, still water, sparkling water

## Abstract

Kidney stone disease (KSD) is a complex disease. Besides the high risk of recurrence, its association with systemic disorders contributes to the burden of disease. Sufficient water intake is crucial for prevention of KSD, however, the mineral content of water might influence stone formation, bone health and cardiovascular (CVD) risk. This study aims to analyse the variations in mineral content of bottled drinking water worldwide to evaluate the differences and describes the possible impact on nephrological and urological diseases. The information regarding mineral composition (mg/L) on calcium, bicarbonate, magnesium, sodium and sulphates was read from the ingredients label on water bottles by visiting the supermarket or consulting the online shop. The bottled waters in two main supermarkets in 21 countries were included. The evaluation shows that on a global level the mineral composition of bottled drinkable water varies enormously. Median bicarbonate levels varied by factors of 12.6 and 57.3 for still and sparkling water, respectively. Median calcium levels varied by factors of 18.7 and 7.4 for still and sparkling water, respectively. As the mineral content of bottled drinking water varies enormously worldwide and mineral intake through water might influence stone formation, bone health and CVD risk, urologists and nephrologists should counsel their patients on an individual level regarding water intake.

## 1. Introduction

Kidney stone disease (KSD), a condition characterized by the formation of crystals within the urinary tract, is a prevalent disease worldwide. Especially in Western countries, hypothetically due to an increase in obesity, diabetes and improved diagnostics, the estimated lifetime prevalence has risen to 14% [1,2,3]. Currently, prevalence rates range from 7–13% in The United States, 5–14% in Europe and 1–5% in Asia [3]. Besides a high risk of recurrence of 53% at 5 years, another factor contributing to the burden of disease is its association with systemic disorders like coronary heart disease, hypertension, diabetes type 2 and osteoporosis. [4,5,6,7,8].

Although KSD has a complex pathophysiology with a multifactorial aetiology, it is important to understand the various processes leading to stone formation to be able to develop a preventive strategy, to reduce precipitation of crystal-forming substances leading to stone formation. The most recognized general intervention regarding primary prevention for stone formation in patients with KSD, regardless of stone composition, is sufficient fluid intake [9,10]. By increasing the urinary output to at least 2 L/day, dilution of stone forming salts occurs, reducing urinary supersaturation. At the same time, stagnation of urine within the urinary tract, a mechanical risk factor for stone formation, is less likely to occur with sufficient diuresis [11,12].

Although the benefit of water therapy was primarily recognized for the prevention of urolithiasis, it seems to be beneficial in other renal diseases as well. A higher water intake is associated with a reduction in cyst growth rate in autosomal dominant polycystic kidney disease (ADPKD) and seems to protect against chronic kidney disease (CKD), and might even slow the progression of CKD [13].

Over time, scientists have investigated the impact of the mineral content of drinking water on our health. Mineral water rich in calcium and bicarbonate for example, provide for an increase in bone mineral density and a decrease in bone resorption [14,15]. Furthermore, magnesium levels in drinking water seem to be inversely related to the risk of death due to coronary heart disease [16].

Regarding KSD, several minerals have been designated as promotors or inhibitors of stone formation. High urinary excretion of calcium, oxalate and uric acid are well known promoters. On the contrary, urinary citrate, potassium and bicarbonate might be protective factors regarding stone formation [17,18,19]. By analysing 24 h urine samples, which is recommended for high-risk stone formers, urine chemistry may reveal such metabolic abnormalities [20].

As sufficient fluid intake seems to be crucial in the prevention of KSD, the question arises as to what fluids to drink. Beverages like soda, lemonade and fruit juices are not recommended due to their high levels of fructose. Although coffee, tea, wine and beer seem to lower the risk for stone formation [21], physicians generally advise their patients to drink water as it is free from caffeine, alcohol and calories. However, we must realize that drinking water may also contain certain minerals that could lead to a rise of urinary stone promotors and inhibitors. Earlier research performed in France, Spain and the USA has already shown a variation in the mineral content of tap and bottled water nationwide [22,23,24]. European studies showed that the mineral composition of commercially available bottled drinking water across Europe varies enormously [25,26]. Possibly, drinking water with certain characteristics could increase stone risk where others might be better in the inhibition of stone formation.

As the consumption of bottled water is increasing worldwide and is not subject to such strict regulations compared to tap water, it is important to gain insight into mineral composition and the possible impact on our health. Therefore, this study aims to analyse the variations in mineral content of bottled ‘still’ and bottled ‘sparkling or carbonated’ water across different manufacturers and countries worldwide to evaluate the differences globally. This study also aims to describe the possible consequences of the mineral composition of drinking water on our general health, with a focus on nephrological and urological diseases.

## 2. Materials and Methods

This descriptive, multi-continental study was conducted to enhance the understanding of the variabilities of mineral content of commercially available bottled drinking water worldwide. The mineral content of bottled still water and bottled sparkling or carbonated water across different manufacturers was analysed globally. For data collection, the information regarding mineral composition was read from the manufacturers’ ingredient label on water bottles which were commercially available in the two main supermarket chains of each country. As an alternative, the online shop of the supermarket could be used. Minerals of interest were bicarbonate, calcium, magnesium, potassium, sodium and sulphates. All data were obtained in milligrams per litre (mg/L) or otherwise converted to mg/L.

The study was conducted in 21 countries worldwide including: Australia, Belgium, Brazil, Canada, France, Germany, Greece, India, Italy, The Netherlands, Poland, Romania, Russia, Saudi Arabia, Serbia, Singapore, Spain, Switzerland, Turkey, The United Kingdom and The United States.

For statistical analysis, the software of SPSS, version 26 (IBM Corp., Armonk, NY, USA), was used. A check for normality showed that the data were not normally distributed, therefore they were treated as non-parametric data. Descriptive statistics and simple boxplots were used to graphically show the distributional features of the data. To improve the visual representation of the data, some extreme values were excluded from the boxplots. The data are available as supplement to the figures.

## 3. Results

For bottled still water, 316 different commercial water brands were analysed. 29 brands (Acqua Panna, Albert Heijn, Aqua, Aquarel, Bar le Duc, Bleu, Cactus, Cano, Chaudfontaine, Contrex, Dassani, Evian, Fiji, Glaceau Smart water, Harrogate, Hépar, Ice Mountain, Life, Meadows, Montcalm, Nestlé PureLife, pH Balancer, pH Infinity, San Benedetto, Solar de Cabras, Vittel, Volvic, Voss, Zagori) were available in up to 11 countries. Table 1 shows the mineral composition (mg/L) of bottled still water by country expressed as median (IQR). Globally, the median mineral content of still water per mineral varies greatly. Median bicarbonate levels for example vary by a factor of 12.6. Calcium levels vary by a factor of 18.7. Median potassium levels did not vary a lot, ranging from 0.7 mg/L to 2.8 mg/L.

**Figure 1:**
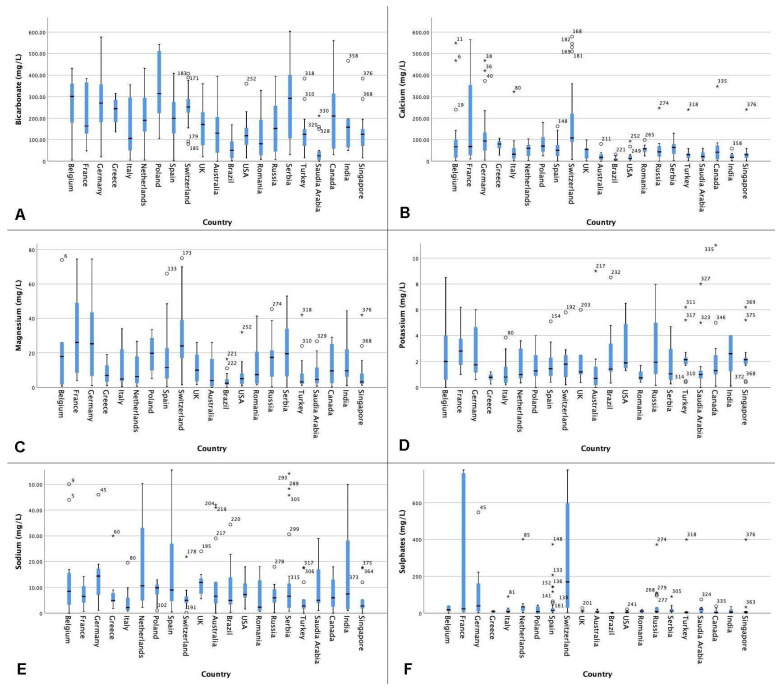
(**A–F**): The mineral composition of bottled still water (mg/L) per mineral by country. ° Outlier. * Extreme value.

Figure 1A–F shows the distribution of the mineral composition (mg/L) of bottled still water worldwide.

Overall, for still water, bicarbonate levels ranged from 0 mg/L (Pureau—Australia, Speyside Glenlivet—Saudi Arabia, Solan de Cabras—Saudi Arabia, Voss—Saudi Arabia) to 2495 mg/L (Heppinger Extra Heil water—Germany) worldwide. Outliers and extreme values for bicarbonate which are excluded in Figure 1 are Sangemini (1010 mg/L), Piwniczanka (1260 mg/L), Zywiec Zdrój (1404 mg/L), Gerolsteiner (1816 mg/L), Staatl. Fachingen Still (1846 mg/L), Heppinger Extra Heil (2495 mg/L). Calcium levels ranged from 0 mg/L (Moores Ultra Pure—Australia) to 579 mg/L (Abdelbodner Cristal—Switzerland). Magnesium levels ranged from 0 mg/L (Moores Ultra Pure—Australia, E’stel—Australia) to 199 mg/L (Heppinger Extra Heil water—Germany). The outliers and extremes were Piwniczanka (87 mg/L), Gerolsteiner (108 mg/L), Eptinger Still (117 mg/L), Abatilles (119 mg/L), Hépar (119 mg/L) and Heppinger Extra Heil (199 mg/L).

Potassium levels ranged from 0 mg/L (Voss—Saudi Arabia/Australia, Spa Reine—Belgium, Żywiec Zdrój—Poland, Harrogate—Saudi Arabia, Dobrowinka—Poland, Contrex—Belgium, Aqua Nordic Naturelle—Germany) to 27.1 mg/L (Aqua Nordic Naturell—Germany). Outliers and extremes excluded in Figure 1 were Piwniczanka (13 mg/L), De L’Aubier (16 mg/L), Staatl. Fachingen Still (16 mg/L), Cristaline (18 mg/L), Heppinger Extra Heil (27 mg/L) and Aqua Nordic Naturelle (92 mg/L).

Sodium levels ranged from 0 mg/L (Jackson Springs—Canada, Dassani—Turkey/Saudi Arabia, Moores Ultra Pure—Australia, Albert Heijn—Belgium, Pureau—Australia) to 564 mg/L (Staatl. Fachingen Still—Germany). For sodium, many outliers and extreme values were identified. Excluded from Figure 1 are Contrex Still (59 mg/L), Aquavia (65 mg/L), Pine Cone Forest (86 mg/L), Perla Covasnei (90 mg/L), Ibira (91 mg/L), Carrefour (95 mg/L), Fontecelto (95 mg/L), Gerolsteiner (118 mg/L), Boni (125 mg/L), Piwniczanka (133 mg/L), Zurzacher Naturelle (154 mg/L), Abatilles (200 mg/L), Saint-Justin (415 mg/L), Heppinger Extra Heil (481 mg/L) and Staatl. Fachingen (564 mg/L).

Sulphates levels ranged from 0 mg/L (Jackson Springs—Australia, Górska Natura—Poland, Dobrowinka—Poland, Żywiec Zdrój—Poland, Nałęczowianka—Poland, Aquarel Nestlé—Poland, Ordal—Belgium, Saint-Justin—Canada) to 190.4 mg/L (Buzias (light)—Romania). Outliers and extremes were Extaler Mineralqual Naturelle (900 mg/L), Carolinen Naturelle (950 mg/L), Contrex Still (1121 mg/L), and Hépar (1530 mg/L).

In total, 224 different commercial water brands were included for sparkling or carbonated water. Seventeen of them (Badoit, Bar le Duc, Cano, Chaudfontaine, Evian Blue, Gerolsteiner, Gerolsteiner Medium, Highland Spring, H-two-O, Nestlé PureLife, Oldenladia, Perrier, San Benedetto, San Pelligrino, Sourcy, Souroti, Voss) were available in up to 10 different countries. Table 2 shows the mineral composition (mg/L) of bottled sparkling or carbonated water by country expressed as median (IQR). As for still water, median levels of the mineral content of sparkling water vary greatly as well, with variations in median bicarbonate levels ranging from 22 mg/L to 1260 mg/L and median magnesium levels varying from 4 mg/L to 53 mg/L.

**Figure 2:**
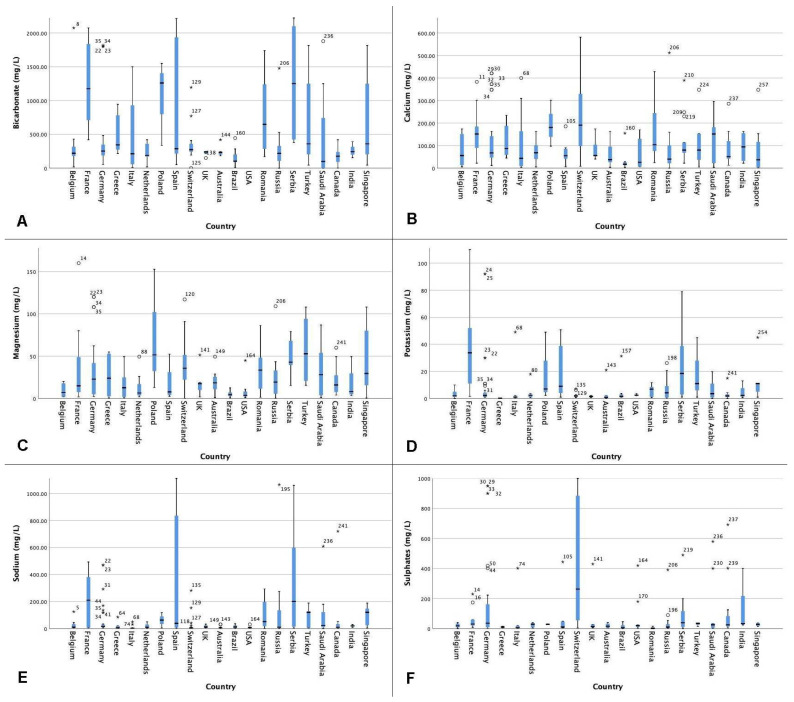
(**A–F**): The mineral composition of bottled sparkling water (mg/L) per mineral by country. ° Outlier. * Extreme value.

Figure 2A–F shows the distribution of the mineral composition (mg/L) of bottled sparkling or carbonated water.

Overall, for sparkling or carbonated water, bicarbonate levels ranged from 0 mg/L (Aqua Mineral—Russia, 365 Days—Russia, San Pellegrino—Saudi Arabia, Voss—Saudi Arabia, Aqua—Saudi Arabia) to 7500 mg/L (Donate Mg—Serbia) worldwide. Outliers and extremes not included in Figure 2 are Borjoni (3754 mg/L), Saint Yorre (4368 mg/L) and Donat Mg (7500 mg/L).

Calcium levels ranged from 0.2 mg/L (Aqua Mineral—Russia) to 581.6 mg/L (Meltinger—Switzerland). Magnesium levels ranged from 0.2 mg/L (Zurzacher Classic—Switzerland) to 1000 mg/L (Donat Mg—Serbia). Donat Mg and Mg Miveia (343 mg/L) are excluded in Figure 2.

Potassium levels ranged from 0 mg/L (Voss—Saudi Arabia/Australia, Nestlé PureLife—Canada, Perrier—Belgium) to 195 mg/L (Ion Water—Singapore).

Sodium levels ranged from 0.3 mg/L (Montana—Saudi Arabia) to 1708 mg/L (Saint-Yorre—France). The outliers and extremes excluded in the boxplot are Donat Mg (1500 mg/L), Borjoni (1590 mg/L) and Saint Yorre (1780 mg/L).

Sulphates levels ranged from 0 mg/L (Ordal—Belgium) to 2200 mg/L (Donat Mg—Serbia). Donat Mg and Lipetsk (1320 mg/L) were the extremes excluded.

A complete overview of the mineral content of all still water brands and sparkling water brands per country, can be found in Table S1, which is submitted as Supplementary Data. 

## 4. Discussion

This descriptive, multi-continental study conducted in 21 countries is, to our knowledge the first study to evaluate the mineral composition of commercially available bottled water worldwide. In total, 316 brands for still water and 224 brands for sparkling water were assessed. Our results show that on a global level the mineral composition of bottled drinkable water varies enormously.

On average, calcium levels of still water vary by a factor of 18.7. Considering each brand individually, a difference of 579 mg/L in calcium content was observed between brands. Moores Ultra Pure—Australia does not contain any calcium, whereas Abdelbodner Cristal—Switzerland for example contains as much as 579 mg/L. This illustrates the wide range in calcium content of commercially available bottled still water worldwide.

Calcium intake plays a significant role in bone homeostasis. A study performed by Costi et al., showed that drinking a mineral water rich in calcium (318 mg/L) significantly contributed to maintaining bone mass of the spine in postmenopausal women [27]. On the other hand, an acidic environment, which can be the result of chronic renal failure or renal tubular acidosis, provokes calcium efflux from the bone, by bone resorption leading to osteoporosis [28]. Adequate calcium intake is therefore of utmost importance for CKD patients. High calcium waters may be a calorie-free nutritional supplement for those with low calcium levels as calcium supplements were thought to increase cardiovascular (CVD) risk [29,30]. However, although the relationship between calcium intake and bone formation is clear, controversy remains whether calcium intake affects the risk for CVD as the evidence is contradictory [31,32,33].

The conception of calcium being a promoter of KSD has long been established. Supersaturation of the urine with calcium, or hypercalciuria, correlates directly to the formation of kidney stones, as a calcium excretion of more than 200 mg/L a day increases stone risk [17]. Consequently, urinary supersaturation of calcium results in a significant risk of recurrence [34]. Although historically a low calcium diet was advised to prevent hypercalciuria, nowadays a normal calcium intake of 1000–1200 mg/day is the standard. A lack of calcium intake through the diet results in a secondary increase in oxalate as calcium binds to oxalate in the gut. Therefore, in case of a low calcium diet, hyper-absorption of free oxalate occurs, resulting in hyperoxaluria [18,35]. A study performed by Curhan et al. showed that a low calcium diet was associated with a 34% higher risk of kidney stones [36]. As 25% of the waters included in our study contain a significant amount of calcium (>100 mg/L), it is important that KSD patients are aware of the calcium content of the water they drink.

Their calcium intake through drinking water should be included as part of the total calcium intake per day and might result in alterations of the patients’ diet.

Another factor contributing to urinary calcium excretion is sodium intake. Since 1964, several studies have documented that an increase in dietary sodium is directly related to calcium excretion, especially in stone formers. An increase in sodium intake of 6 g/day, could lead to an increase in calcium excretion of 40 mg/day [37]. Furthermore, hypercalciuria was corrected in approximately 30% of idiopathic hypercalciuria patients by following a low sodium diet [38]. This phenomenon can be explained by the renal handling of sodium and calcium. Reabsorption of calcium in the distal renal tubule is dependent on sodium exchange. A high sodium load will therefore result in increased urinary calcium. Secondly, hypervolemia induced by a high sodium load might alter sodium and calcium reabsorption [37,38,39].

Although median sodium levels were generally low, our study did include bottled water with high sodium content. For 9 water brands, sodium levels exceeded 1000 mg/L (still water: Element—Serbia (1605 mg/L), sparkling water: Heba Strong—Serbia (1060 mg/L), Lipetsk—Russia (1065 mg/L), San Narciso—Spain (1080 mg/L), Vichy Catalan—Spain (1097 mg/L), Malavella—Spain (1115 mg/L), Donat Mg—Serbia (1500 mg/L), Borjomi—Russia (1590 mg/L), Saint-Yorre—France (1708 mg/L)). By drinking 3 L of such water, KSD patients might unintentionally increase the risk for stone formation by inducing hypercalciuria as their sodium intake, which often already exceeds the recommended daily intake, significantly increases. However, also for non-stone formers, monitoring the sodium intake is relevant as a high sodium intake of more than 5 g/day is associated with high blood pressure and significantly related to a higher risk of stroke and of end-stage kidney disease, particularly when KSD has contributed to CKD development [40].

Contrary to calcium and sodium, bicarbonate may protect against kidney stone formation. Bicarbonate as an alkaline substance increases urinary pH and stimulates citrate excretion, an inhibitor of stone formation. Earlier studies have demonstrated that consuming a mineral water rich in bicarbonate (>1715 mg/L) significantly increases urinary pH to metaphylactic levels around 6.7 [41,42]. Furthermore, the excretion of citrate, which chelates urinary calcium to form soluble complexes and also prevents aggregations of calcium oxalate, significantly increased to levels normally reached by pharmacologic treatment with sodium potassium citrate [41]. This suggests that mineral water instead of (or in combination with) pharmacologic agents could be used as a metaphylaxis therapy.

There are several water brands included in this study with such a high bicarbonate content (> 1715 mg/L), most of them being sparkling water (22 sparkling waters, three still waters). Some of these even contain extreme amounts of bicarbonate, with concentrations up to 7500 mg/L (Donat Mg—Serbia). However, a study performed by Karagülle et al. demonstrated that the ingestion of bicarbonate water with a content of 2673 mg/L also increased urinary supersaturation with calcium phosphate. Alkaline waters are not suitable for phosphate stone formers as the goal is to lower urinary pH in such patients [42].

Another mineral potentially inhibiting stone formation is magnesium. Like bicarbonate, magnesium provides for an alkali load resulting in more alkaline urine. Moreover, magnesium competes with calcium in binding to free oxalate, which increases solubility. Therefore, theoretically, magnesium can reduce oxalate reabsorption in the gut and the urinary tract to prevent precipitation of calcium oxalate [43]. However, controversy remains as several studies failed to demonstrate a decline in urinary oxalate in case of higher magnesium intake where other studies did [43].

Magnesium is a key nutrient in several biochemical processes in the body. It is involved in glucose homeostasis, lipid metabolism, neuronal functioning, bone metabolism and many more cellular processes throughout the human body [44]. Many studies have been performed to evaluate the effects of dietary magnesium on our health, including ischemic heart disease, diabetes type 2, hypertension and CKD [44]. Considering CVD risk, studies have shown that dietary magnesium is inversely related to CVD risk and fatal ischemic heart disease [16,45,46]. This also applies for patients with CKD, who are already at increased risk for cardiovascular mortality [47]. A meta-analysis performed by Gianfredi et al., evaluated the association of magnesium and calcium rich drinking water (hard water) with CVD risk. Although heterogeneity was present, the consumption of hard water could be protective regarding CVD risk [48].

Adequate potassium intake also lowers CVD risk and high potassium intake might even counterbalance for the CVD risk associated with high sodium intake. As potassium is mainly found in vegetables and fruits, this correlation might be explained by a healthy diet overall lowering cardiovascular risk [40,46].

Regarding KSD, potassium intake is inversely related to KSD risk [36]. A study performed by Ferraro et al. showed that a daily potassium intake of 2781 mg/day lowers the risk of kidney stones by 33–56% [49]. As the water currently studied did not contain as much potassium, KSD patients should predominantly rely on vegetables and fruits to achieve an adequate potassium intake.

With the increasing prevalence of KSD the management should shift more towards focusing on the prevention of recurrence. Although pharmacologic treatment with thiazide diuretics and potassium citrate is well established in the current guidelines, modification of the diet for the prevention of KSD is gaining interest [50,51]. As fluid therapy is the corner stone in the prevention of stone formation, urologists should realize that drinking water contains minerals that could affect urinary metabolites either promoting or inhibiting kidney stone formation. Furthermore, as this study shows, the mineral composition of bottled drinking water varies greatly worldwide. Therefore, effective dietary counselling on the prevention of stone recurrence should also include advice on what type of water to drink considering stone composition. Also, the differences in mineral composition between tap water versus bottled water should be addressed. Although the mineral composition of tap water does vary locally, it does not vary to such extent that it affects stone development as tap water is strictly regulated by the government. However, as shown by our study, the mineral composition of bottled water does vary enormously worldwide. Although most countries have access to tap water, the consumption of bottled water is increasing worldwide. Especially in Western countries, where good quality tap water is easily accessible, this seems paradoxical. In the US for example, the average consumption per capita has doubled to 138.17 L in 2015 [52]. In France, the consumption of bottled water per capita increased from 6 L per person in 1940 to 141 L per person in 2015, a 2350% increase [53]. Although more people are gaining access to clean tap water, a trend towards bottled water also occurs in developing countries [54].

Besides the importance of knowledge on the mineral composition of water for KSD patients to prevent stone formation, an adequate dietary mineral intake, which can be supplemented by drinking mineral water, is essential for bone health and lowering CVD risk. Although the biochemical processes in our body involving minerals like calcium, bicarbonate and magnesium are complex, maintaining a low-grade metabolic alkalosis might protect against age-related diseases as these seem to be related to acidosis [55].

To the best of our knowledge, this is the first study to analyse the mineral composition of commercially available bottled still and bottled sparkling or carbonated water worldwide. As earlier studies performed in Europe showed previously [25,26], this global study shows that the mineral composition of bottled water varies greatly worldwide. We intended to analyse the mineral content of bottled water worldwide and took samples from 21 countries. A limitation of our study is that we relied on information given by the manufacturers on the labels regarding the mineral composition of the included waters rather than independent laboratory measurements. Unfortunately, our study did not include bottled drinking water from the African continent. Also, we did not evaluate the mineral composition of tap water. It would be interesting to investigate to what extent the mineral composition of tap water differs from that of bottled drinking water globally. Secondly, it would be interesting to compare geographical differences in the mineral composition of tap water to KSD prevalence rates, CVD risk and osteoporosis worldwide. However, as there are lots of other dietary and non-dietary factors contributing to the risk of stone formation, CVD and bone health, it will be difficult to determine the exact role of the mineral composition of water on the development of disease.

## 5. Conclusions

KSD is a complex and multifactorial disease with increasing prevalence rates worldwide. As recurrences rates are high, the focus in management of this disease has to include strategies of prevention. Although drinking sufficient amounts of water is recommended, drinking water can contain inhibitors as well as promotors of stone formation. On the other hand, adequate dietary mineral intake is important for bone health and lowers CVD risk. As the mineral content of bottled still and bottled sparkling or carbonated water varies enormously across the globe, urologists and nephrologists should counsel their patients on an individual level regarding their water intake.

## Figures and Tables

**Table 1:** The mineral composition (mg/L) of bottled still water expressed as median (IQR).

Country	Mineral Composition (mg/L)					
	Bicarbonate	Calcium	Magnesium	Potassium	Sodium	Sulphates
Australia	130.00 (34.00–258.00)	18.00 (6.40–31.95)	3.95 (0.525–16.50)	0.70 (0.17–1.60)	6.60 (3.79–12.00)	6.55 (3.40–14.00)
Belgium	301.00 (180.00–360.00)	66.80 (16.50–101.00)	18.00 (1.80–26.00)	2.00 (0.60–4.00)	8.50 (3.25–15.60)	18.00 (10.00–40.00)
Brazil	50.71 (13.20–95.49)	5.78 (3.43–13.30)	2.42 (1.55–4.71)	1.42 (1.22–4.00)	4.95 (3.34–13.93)	1.56 (0.93–2.95)
Canada	210.00 (35.00–330.00)	42.00 (7.00–73.00)	9.60 (2.50–25.15)	1.30 (1.00–3.00)	6.00 (2.48–13.00)	4.10 (1.50–12.55)
France	163.50 (127.00–372.00)	68.00 (19.00–468.00)	26.00 (8.00–56.00)	2.80 (1.60–4.00)	6.50 (3.00–11.60)	24.00 (8.10–1121)
Germany	270.00 (182.00–356.50)	94.00 (47.00–142.00)	25.25 (6.65–43.50)	1.75 (1.15–4.65)	14.40 (7.10–17.30)	39.55 (9.00–162.00)
Greece	244.00 (182.00–286.00)	79.65 (60.00–93.10)	7.00 (3.30–12.80)	0.79 (0.60–1.00)	4.90 (4.35–7.80)	9.15 (5.00–14.00)
India	158.50 (64.00–196.80)	17.00 (13.60–33.60)	9.65 (6.20–22.00)	2.60 (0.50–4.00)	7.45 (1.55–28.2)	6.00 (3.20–19.30)
Italy	106.00 (50.00–296.00)	32.20 (11.80–60.36)	4.90 (3.70–22.10)	0.80 (0.35–1.60)	2.20 (1.00–6.00)	8.60 (6.00–22.00)
The Netherlands	190.00 (106.00–305.00)	60.00 (15.00–80.00)	6.25 (2.46–18.00)	1.00 (0.60–3.30)	10.60 (4.80–36.20)	34.00 (10.00–40.00)
Poland	314.45 (223.40–512.45)	70.13 (43.85–111.20)	19.75 (9.92–28.55)	1.28 (0.89–2.50)	9.85 (7.28–11.05)	7.94 (0.00–36.25)
Romania	81.11 (28.00–192.03)	57.85 (43.50–62.77)	7.50 (2.21–20.60)	0.75 (0.40–1.70)	2.33 (0.93–12.74)	10.70 (2.10–19.29)
Russia	152.00 (45.00–258.00)	43.30 (21.20–70.60)	17.40 (6.22–21.40)	1.95 (1.03–5.00)	5.96 (4.10–9.29)	8.50 (6.12–31.00)
Saudi Arabia	25.00 (6.10–50.00)	21.50 (12.00–40.50)	4.70 (2.00–13.00)	1.00 (0.70–1.40)	5.00 (3.80–17.00)	21.80 (4.00–30.00)
Serbia	292.5 (106.00–400.80)	64.01 (33.82–79.90)	19.50 (6.50–34.00)	1.05 (0.59–2.96)	6.60 (2.10–11.50)	11.55 (7.15–23.00)
Singapore	125.00 (71.00–150.00)	30.50 (15.00–37.10)	3.20 (2.10–8.00)	2.15 (1.80–2.30)	2.80 (1.80–5.20)	6.00 (3.00–9.10)
Spain	199.30 (129.20–275.00)	50.79 (24.25–75.25)	11.50 (5.00–23.40)	1.45 (0.90–2.30)	9.00 (4.70–27.00)	14.40 (8.10–26.75)
Switzerland	252.00 (226.30–289.00)	108.00 (89.00–221.00)	24.00 (17.00–39.00)	1.80 (0.80–2.50)	5.00 (4.00–6.50)	170.00 (29.50–597.00)
Turkey	125.00 (71.00–150.00)	30.50 (15.00–37.10)	3.20 (2.10–8.00)	2.15 (1.80–2.30)	2.80 (1.80–5.20)	4.50 (2.90–8.60)
The United Kingdom	171.00 (74.00–240.00)	55.00 (12.00–59.00)	10.05 (3.50–19.00)	1.20 (1.00–2.50)	11.90 (7.03–15.00)	12.00 (9.00–14.00)
The United States	118.50 (76.00–155.00)	12.00 (8.70–26.20)	5.05 (2.10–8.05)	1.90 (1.50–4.90)	7.25 (6.15–11.55)	5.65 (3.80–10.00)

**Table 2:** The mineral composition (mg/L) of bottled sparkling water expressed as median (IQR).

Country	Mineral Composition (mg/L)					
	Bicarbonate	Calcium	Magnesium	Potassium	Sodium	Sulphates
Australia	233.00 (200.00–243.00)	37.75 (25.98–95.60)	19.00 (4.00–29.00)	1.00 (0.00–2.00)	7.00 (1.90–10.00)	16.00 (6.00–33.00)
Belgium	22.00 (180.00–317.00)	56.00 (13.50–151.50)	7.00 (2.00–18.00)	2.00 (1.00–5.00)	10.60 (9.00–33.30)	19.00 (8.00–33.00)
Brazil	102.84 (91.41–203.28)	17.14 (13.90–26.05)	4.00 (3.00–7.00)	1.00 (1.00–3.00)	11.80 (3.98–23.02)	6.00 (2.00–38.00)
Canada	176.60 (77.00–243.00)	51.00 (42.00–150.00)	16.00 (6.00–29.00)	2.00 (1.00–4.00)	10.00 (6.00–36.10)	25.00 (11.00–125.00)
France	1175.00 (710.00–1837.00)	151.50 (90.00–185.00)	15.00 (8.00–49.00)	34.00 (11.00–52.00)	210.00 (7.47–381.00)	30.00 (20.00–59.00)
Germany	253.00 (189.00–349.00)	67.50 (47.00–142.00)	23.00 (5.00–42.00)	2.00 (1.00–4.00)	15.80 (13.30–29.90)	36.00 (9.00–162.00)
Greece	344.15 (274.00–781.00)	87.20 (59.30–188.00)	24.00 (3.00–53.00)	0.00 (0.00–0.00)	6.02 (4.43–20.00)	11.00 (5.00–12.00)
India	243.00 (155.00–390.00)	94.30 (3.65–155.65)	8.00 (5.00–30.00)	2.00 (1.00–13.00)	20.00 (9.00–31.20)	33.00 (24.00–402.00)
Italy	212.55 (57.40–930.00)	43.50 (9.10–164.00)	13.00 (2.00–25.00)	1.00 (1.00–2.00)	3.07 (1.50–6.00)	6.00 (4.00–18.00)
The Netherlands	190.00 (170.00–360.00)	68.50 (40.90–101.50)	7.00 (3.00–18.00)	2.00 (1.00–3.00)	10.30 (6.00–30.60)	29.00 (9.00–37.00)
Poland	1260.00 (335.60–1550.00)	180.90 (97.80–301.00)	52.00 (13.00–153.00)	7.00 (2.00–49.00)	63.00 (4.59–118.00)	29.00 (27.00–32.00)
Romania	648.00 (244.00–1364.50)	104.00 (74.85–252.60)	34.00 (11.00–49.00)	7.00 (1.00–9.00)	51.40 (15.41–205.00)	1.00 (1.00–16.00)
Russia	218.50 (107.00–330.00)	40.20 (21.60–101.00)	19.00 (6.00–33.00)	4.00 (1.00–9.00)	10.41 (4.90–135.00)	10.00 (5.00–30.00)
Saudi Arabia	100.00 (0.00–744.00)	151.50 (22.00–182.00)	28.00 (4.00–54.00)	4.00 (1.00–11.00)	23.50 (9.60–122.00)	25.00 (5.00–35.00)
Serbia	1251.00 (423.00–2100.00)	80.00 (67.84–114.00)	43.00 (40.00–68.00)	19.00 (3.00–39.00)	200.70 (14.10–598.00)	39.00 (11.00–116.00)
Singapore	360.00 (205.00–1250.00)	37.10 (1.00–153.00)	30.00 (16.00–80.00)	11.00 (5.00–11.00)	120.50 (24.75–148.00)	28.00 (18.00–37.00)
Spain	287.00 (215.50–1935.50)	55.00 (32.00–86.80)	8.00 (4.00–31.00)	9.00 (3.00–49.00)	38.80 (7.55–835.50)	11.00 (7.00–48.00)
Switzerland	273.50 (243.50–360.50)	191.00 (97.70–330.00)	36.00 (22.00–52.00)	2.00 (1.00–3.00)	5.20 (4.00–7.00)	263.00 (55.00–885.00)
Turkey	360.00 (205.00–1250.00)	80.00 (37.10–153.00)	53.00 (21.00–94.00)	11.00 (6.00–28.00)	120.50 (6.50–128.00)	35.00 (14.00–38.00)
The United Kingdom	240.00 (215.00–245.00)	56.00 (55.00–104.00)	18.00 (10.00–19.00)	2.00 (1.00–2.00)	11.50 (7.47–24.00)	13.00 (9.00–28.00)
The United States	n.a.	25.95 (6.65–130.00)	4.00 (2.00–8.00)	2.00 (2.00–4.00)	8.30 (3.30–11.00)	20.00 (11.00–26.00)

## Data Availability

Not applicable.

## Supplementary Materials

The following are available online at https://www.mdpi.com/article/10.3390/jcm10132807/s1, Table S1: The mineral composition (mg/L) of bottled still and sparkling water by country.
