# Supplementary material for: Global Variations in the Mineral Content of Bottled Still and Sparkling Water and a Description of the Possible Impact on Nephrological and Urological Diseases
Source: J Clin Med. 2021 Jun 27;10(13):2807. doi: 10.3390/jcm10132807 (PMC8267898; doi:10.3390/jcm10132807)
Supplement: Supplementary file 1 [file jcm-10-02807-s001.zip › jcm-1253161-supplementary.pdf]

Table S1: The mineral composition (mg/L) of bottled still and sparkling water by country

Water brands per country		Mineral composition (mg/L)					
		Bicarbonate	Calcium	Magnesium	Potassium	Sodium	Sulphates
**EUROPE**							
**Belgium**							
Still	Albert Heijn		10.4			0	
	Bar le Duc	170	47			10.6	
	Boni	345	59			59	
	Bru	180	21	20	2	8	19
	Chaudfontaine	305	65	18	2	44	40
	Contrex	372	468	74	0	9	1121
	Everyday Aurelie		106			3.5	
	Everyday CristalRoc	200	73			4.5	
	Everyday Louise	432	66			50	
	Evian	360	80	26	1	6	12
	Hépar	383.7	549	119	4	14.2	1530
	Montcalm	5.2	3	0.7	0.6	2.2	10
	Nestlé PureLife		70			2	
	Ordal	317	143	7	5	17	0
	Rocheval	297	96			10.6	
	Spa reine	15	4	1	0	3	4
	Val	220	6	1.8	8.5	125	18
	Valvert		67.6			1.9	
	Vittel		240			5.2	
	Volvic		12			12	
Sparkling	Spa Bruis	18	6	2	1	5	8
	Chaudfontaine	305	65	18	2	44	40

		Bru	180	21	20	2	8	19
		Ordal	317	143	7	5	17	0
		Val	220	6	2	10	125	18
		Bar le Duc	170	47			10.6	
		Sourcy	180				10	
		San Pelligrino		174			33.3	
		Perrier	430	160		0	9	33
**France**								
	Still	Abatilles	127	19	9	4	100	8
		Aix Les Bains	337	68	35	1.6	9	61
		Celtic	48	10.5	4	1.9	1.1	6
		Contrex	372	468	74.5	2.8	9.4	1121
		Courmayeur	130	565	56	2.8	0.6	1477
		Evian	360	80	26	1	6.5	14
		Hépar	383.7	549	119	4.1	14.2	1530
		Ondine	163.5	46.1	4.3	3.5	6.3	9
		Vittel	384	240	42	1.9	5.2	400
		Volvic	71	11.5	8	6.2	11.6	8.1
		Wattwiller	135	35	11	1	3	24
	Sparkling	Badoit	1250	153	80	11	180	35
		Chateldon	2075	383	49	35	240	20
		Orezza	710	185	16.5	1.55	6.9	14
		Perrier	420	150	3.9	<1	9.6	25.3
		Rozana	1837	301	160	52	493	230
		Sainte Marguerite	812	71	40	33	302	59
		Saint-Yorre	4368	90	11	110	1708	174
		Salvetat	530	160	7.5	2	5	20
		Vals	1100	22.2	13.5	33.8	381	45.1

		Vichy Celestin		104	1.8		7.47	9.01
**Germany**								
	Still	Adelholzener Naturell	431	94.7	30.4	0.6	4.6	6.9
		Aqua Nordic Naturell	195	92.0	3.7	0	19	
		Bad Pyrmonter Naturell	253	53	24.5	0.9	5.2	9
		Black Forest	30.5	6.7	2.6	1.7	1.1	2.9
		Carolinen Naturelle	317	421	43	4.3	14.8	950
		Christinen Carat Naturelle	282	106	8.4	1.5	11.4	61
		Contrex	372	468	74.5		9.4	1121
		Evian	360	80	26	1	6.5	14
		Extaler Mineralquell Naturell	266	373	62.1	1.6	10.8	900
		Gerolsteiner Naturell	577	125	44	5	17	24
		Heppinger Extra Heil Water	2495	150	199	27.1	481	80
		Ja! Mineral water Still 0.5L	189	67.5	12.5	1.1	15.2	59
		Ja! Mineral water Still 1.5L	109	9.4	3	1.4	97.7	40.1
		Rewe Beste Wahl Sport	296	235	66	3.1	46	548
		Rewe Beste Wahl Still	353	142	32.4	2.8	15	162
		San Benedetto	296	51.4	29.7	0.97	6	4.2
		Share	274	39.7	21.2	1.2	17.6	8.1
		Staatl. Fachingen	1846	98.7	59.2	16.1	564	39
		Vilsa Naturelle	175	47	3.6	2	16.4	10
		Vio	152	51	5.3		15	19
		Vittel	248	94	20		7.7	120
		Volvic Naturelle	74	12	8	6	12	9
		Vöslauer Ohne	249	114.4	40.9	1.8	14	223
		Voss	20	5	1		6	
	Sparkling	Adelholzener Classic	345	70.9	32	1.1	13.3	28
		Adelholzener Sanft	345	70.9	32	1.1	13.3	28

Apollinaris Classic	1800	90	120	30	470	100
Apollinaris Medium	1800	90	120	30	470	100
Aqua Nordic Classic	195		3.7	92	19	
Aqua Nordic Medium	195		3.7	92	19	
Bad Pyrmonter Classic	253	53	24.5	0.9	5.2	9
Bad Pyrmonter Light Pearly	253	53	24.5	0.9	5.2	9
Bad Pyrmonter Medium	253	53	24.5	0.9	5.2	9
Carolinen Classic	317	421	43	4.3	14.8	950
Carolinen Medium	317	421	43	4.3	14.8	950
Christinen Carat Spritzig	483	53	4.7	9.4	292	120
Extaler Mineralquell Classic	266	373	62.1	1.6	10.8	900
Extaler Mineralquell Medium	266	373	62.1	1.6	10.8	900
Gerolsteiner Medium	1816	348	108	11	118	38
Gerolsteiner Sprudel	1816	348	108	11	118	38
Graf Rudolf Quelle fresh	456	107	11	2.5	22.1	1.1
Graf Rudolf Quelle mild	456	107	11	2.5	22.1	1.1
Hella Mineral Water Classic	155	62	2.3		7.2	39
Hella Water	155	62	2.3		7.2	39
Ja! Classic 0.5L	189	67.5	12.5	1.1	15.2	59
Ja! Classic 1.5L	118	24.9	7.4	2.2	136	34.1
Ja! Medium 0.5L	189	67.5	12.5	1.1	15.2	59
Ja! Medium 1.5L	56	17.6	5.8	1.3	34	25.1
Mineau Classic	408	142	51.6	3	170	418
Mineau Medium	203	41.1	3.2	3	29.9	4
Rewe Beste Wahl Classic	353	142	32.4	2.8	15	162
Rewe Beste Wahl Medium	353	142	32.4	2.8	15	162
San Benedetto Elite Prickelend	296	51.4	29.7	0.97	6	4.2
San Pellegrino Medium	243	164	49.5	2.2	31.2	402

	Share Medium	274	39.7	21.2	1.2	17.6	8.1
	Share Prickelend	274	39.7	21.2	1.2	17.6	8.1
	Vilsa Classic	175	47	3.6	2	16.4	10
	Vilsa Leichtperlig	175	47	3.6	2	16.4	10
	Vilsa Medium	175	47	3.6	2	16.4	10
	Vio Medium	152	51	5.3		15	19
	Volvic Leightperlig	74	12	8	6	12	9
	Vöslauer Mild	249	114.4	40.9	1.8	14	223
	Vöslauer Pricklend	249	114.4	40.9	1.8	14	223
**Greece**							
Still	Ab	236.25	79.65	4.24	0.87	4.35	15.53
	Arethousa	286	64	19		7.8	6
	Avra	182	60	8.9	1.2	9	14
	Dirfys	286	64	19		7.8	6
	Erymanthos	308	95.5	8.5	1	30	14.7
	Korpi	314.8	106.5	3.3	0.68	4.7	5
	Marata	236.25	79.65	4.24	0.87	4.35	15.53
	Samaria	160	31	14	0.5	7	5
	Theoni	149	42	1	0.26	1.8	5
	Vikos	270	93.1	1.9	0.7	2.6	12.2
	Yas	250	98	7		4.9	11
	Zagori	244	83	3.06	1.02	2.85	9.15
	Zaros	135	28.2	12.8	0.6	6.7	5
Sparkling	Ab	384	45	53		20	16
	Ioli Fizzy	274	59.3	20.8		7.33	10.5
	Korpi Fyssalis	304.3	100.8	2.5		4.7	5
	Mitsikeli Vikos	215	73.6	0.5		1.4	5
	Souroti	781	188	55	0.2	85	

		Xino Nero Florinas	945.81	233.9	27.2		4.43	12.2
**Italy**								
	Still	Acqua Panna	106	32.2	6.5	0.8	6.6	22
		Chiarella	217.8	40.6	22.1	0.2	0.9	8.6
		Lauretana	5	1.5	0.42	0.19	1	
		Levissima	56.8	19.9	1.7	1.6	2.1	16.9
		Norda	50	11.8	3.7	0.82	2.2	6
		Pejo	54	19	4.9	1.9	2.2	27.2
		Primula	355	91	33.2	0.78	9.8	
		Rocchetta	185.4	60.36	3.73	0.35	3.87	7.54
		Rosalpe	27	5.6	3.9	0.24	0.8	
		San Benedetto	296	51.4	29.7	2.97	6	4.2
		San Bernardo	31	10.2	0.48	0.48	0.8	1.5
		Sangemini	1010	323	16.5	3.85	19.6	
		Vitasnella	324	95	34	1.5	3.7	91
	Sparkling	Acqua Pam		3.7	0.6	0.56	1.3	3.6
		Brio Rossa Rochhetta	182.1	57.36	3.23	0.35	4.13	6.75
		Ferrarelle	1500	400	25	49	50	4.8
		Lete	930	310	13.2	1.82	5.1	
		Levissima Frizzante	57.4	20.4	1.8	1.6	1.9	16.1
		Nestle Vera	147	35.6	12.7	0.5	2	19.9
		San Benedetto	296	51.4	29.7	0.97	6	4.2
		San Bernardo	27.9	9.1	0.6	0.4	0.8	2.6
		San Pellegrino	243	164	49.5	2.2	31.2	402
		Sant'Anna	10	2.9			1.5	
**the Netherlands**								
	Still	Acqua Panna	106	32.2	6.5	0.8	22	22
		Albert Heijn	360			2.7	5	

	Albert Heijn Basic	280	104	3.7	1.8	3.7	52
	Bar le Duc	170	47	3.4	0.6	10.6	<1
	Chaudfontaine	305	65	18	2.5	44	40
	Cristaline	432	66	26	18	50	34
	Dalphin	212	71	6.5	3.3	30	37
	Evian	360	80	26	1	6.5	14
	Jumbo	190	97	10.8	3.4	18.5	
	Just Water		15	2.35	0.35	50.4	35
	Montcalm	5.2	3	0.7	0.6	2.2	10
	Natural Cool		89.5		3.6	36.2	
	Solan de Cabras	284	60	26.7	1	4.8	
	Sourcy	180	49	6	1	10	10
	Spa	17	5	2	0.5	3	4
	Tavina Elegantia	36.5	7.86	2.46			
Sparkling	Albert Heijn	360			2.7	5	
	Albert Heijn Basic	257	98	2	0.6	3.1	33
	Bar le Duc	170	47	3.4	0.6	10.6	<1
	Chaudfontaine	305	65	18	2.5	44	40
	Cristaline		66	26	18	50	
	Dalphin	212	71	6.5	3.3	30	37
	Gerolsteiner Medium	181.6	34.8	10.8	1.1	11.8	3.8
	Hébron	360	105	16	3	6	41
	Jumbo	190	97	10.8	3.4	18.5	
	Jumbo Slightly sparkling	367	142	20	3	6	
	Natural Cool		89.2		3.6	39.2	
	Perrier	420	150	3.9	<1	9.6	25.3
	San Pelligrino		164	49.5	2.2	31.2	
	Sourcy	180	49	6	1	10	10

		Spa Finesse	70	11	5.5	1	9	8.5
		Spa Intense	18	5.5	1.9	0.5	5	7.5
		Tavina Elegantia Vivace	36.5	7.86	2.46			
**Poland**								
	Still	Aquarel Nestle	482.3	112.2	24.3	4	13	0
		Arctic	260.1	74.15	13.37	1.35	8.12	25.64
		Cisowianka	542.6	131.2	22.48	0.78	10.71	2.58
		Dobrowinka	276.9	58.12	33.42	0	2	0
		Górska Natura	104	25.2	6.47	1.93	1.04	0
		Jurajska	329.9	66.1	32.8	2.2	10	40.5
		Kropla Beskidu	186.7	44.09	17.01	1	11.1	43.62
		Mama I Ja	165	43.6	5.05	1.06	9.7	13.3
		Nałęczowianka	453.7	110.2	23.1	2.8	11	0
		Piwniczanka	1260	180.8	87	13	133	32
		Ustronianka	299	98.1	16.52	1.21	6.44	41.8
		Żywiec Zdrój	1403.7	41.69	5.62	0	9.65	0
	Sparkling	Kinga Pienińska	335.6	97.8	13.13	2.3	4.59	28.5
		Muszynianka	1260	180.9	152.7	7	63	32
		Staropolanka	1550	301	51.6	48.9	118	27.3
**Romania**								
	Still	Buzias (light)		74.85	28.2		190.4	
		Herculane		61.53	3.06		0.62	
		Apa Craiului		62.8	1.5		2.96	
		Keia		43.8	2.92		1.44	
		Perla Covasnei		24.8	11.8		90	
		Aqua Carpatica	193	45.8	13		0.8	
		Azuga	161	43.2	6.29	0.75	2.59	
		Borsec	329	55.1	32.8		2.07	

	Dorna	191.05	62.73	1.46	0.4	0.93	
	Bucovina	225.7	60.6	8.7	1.7	7.4	19.29
	Aquatique	112.21	33.69	1.46		0.93	<16
	Carpatina (light)		98.51	41.35		18.08	
Sparkling	Buzias		74.85	28.2		190.4	
	Apa Craiului		72.1	1.41		1.15	
	Stanceni		86.71	38.25		22.55	
	Lipova		101.9	33.63		109.2	
	Carpatina (forte)		104.1	44.18		15.41	
	Poiana Negri (apriga)		254.6	48.56		218	
	Poiana Negri (cumpatata)		252.6	48.56		218	
	Biborteni		265.5	86.15		213.4	
	Valea Izvoarelor		47.02	9.66		93	
	Bucovina		155.2	48.04		20.3	
	Aqua Carpatica	171	41.2	11.4		1.39	
	Perla Apusenilor	1738.5	428.5	66.23	6.85	47.21	0.53
	Perla Harghitei	648	104	39.3	8.56	51.4	1.27
	Tusnad	1364.5	236.24	78.65	11.75	293.75	
	Poiana Negri	1116.9	217.8	27.4		205	
	Zizin	244	77.1	3112	1.09	8.81	<40
	Perenna Premier	327.3	94.9	7.7	0.57	1.76	15.8
	Dorna		300.1	12.51		21.5	
	Perla Covasnei		24.8	11.8		90	
**Russia**							
Still	Chernogolovskaya	245	55.3	21.4	4.34	4.7	8.5
	Pine cone forest	171	0.92	0.28	0.56	86.4	12.5
	Bon aqua	9	21.2	21.1	0.17	2.93	103
	Aqua mineral	7	0.21	8.32	0.76	9.01	31

	Saint spring	82	25.7	7.44	0.46	4.10	6.8
	Evian	355	85.1	27.3	1.09	7.03	13.2
	Senezhskaya	351	70.4	29.1	11.3	5.96	5.4
	Baikal	67	17.5	3.44	1.03	3.85	5.4
	Vittel	395	247	45.4	2.42	5.48	373
	Svetla	383	75.7	38.9	1.33	5.48	11.5
	Nestle PureLife	45	24.9	6.22	12.7	11.2	<0.5
	Dzhigem	152	76.6	17.4	1.95	9.02	95.5
	Okay	146	43.3	19.9	4.51	6.12	55
	What you need	272	81.9	37.7	7.98	10.5	111
	Glaceau Smartwater	11	70.6	5.58	5.34	0.16	<0.5
	Arhiz	165	32.5	12.4	1.7	9.29	7.1
	SelterS	258.15	67.1	20.7	2.35	8.86	27
	Novoterskaya	34	19.8	2.25	1.03	2.56	7.9
Sparkling	Pine cone forest	330	101	30.8	1.92	17.7	24.6
	Pine cone forest 2	125	1.1	0.28	0.54	84.8	13
	Bon aqua	243	35.6	20.7	0.2	135	3.2
	Aqua mineral	0	0.22	7.95	0.74	10.9	30
	Saint spring	31	22.2	6.54	0.39	3.52	6.4
	Lipetsk	209	106	39.7	20.6	1065	1320
	Seven streams	528	13.2	4.51	11.6	274	90
	Perrier	395	160	4.25	0.49	10.1	17.2
	Borjomi	3754	142	43.5	26.2	1590	4
	Jigem	123	64.3	14.7	1.85	16.8	81
	Arhiz	144	28.5	11.0	1.5	8.81	7.1
	What you need	228	77.5	35.4	7.56	9.91	54
	Okay	107	40.2	18.1	4.16	5.96	2.7
	Senezhskaya	308	54.0	23.0	8.58	4.62	5.4

		Bonvida	325	67.6	27.8	6.96	4.9	4.5
		365 days	0	0.58	0.28	0.30	15.8	<0.5
		Narzan	1478	512	109	9.14	195	391
		Pilgrim	83	21.6	3.47	1.25	8.46	10.1
**Serbia**								
	Still	Glaceau Smart water		65	4.8	4.7		
		Vlasinska Rosa	42.7	9.6	0.82	1	2.7	5.2
		Jana	381	63.8	32	0.6	1.8	7.2
		Aqua Viva	314	90	13	1.8	11.5	24
		Sara	254.4	48	25.3		2.1	10
		Prolom voda	74.5	3	0.1	0.5	48.3	2.5
		Radenska	280	56	20	0.8	7.6	1
		Ukusi moga kraja	400.8	66.2	39.5	1.1	4.56	20.4
		Vlasina	30.5	7.42	4.27		1.4	1.31
		Vrnjci	451.2	64.21	36.88	4.41	54.21	39.12
		Golijska ledena	79.3	20.44	3.64	0.27	0.79	8.52
		Raj	451	123	19	0.5	3.9	11.7
		Jazak	423	80	41.8	3.8	6.1	24.7
		Saint John`s	377.2	78.1	21	1.28	7.5	11.4
		Tronoša		85	39.3	0.579	1.67	
		Heba Natural	225.7	56.1	14.9	3.84	30.6	43
		Aqua Gala	402	62.1	34	2	14.4	13
		Voda Voda	305	75.6	14.9	2.12	10.6	7.1
		Element	134.2	33.82	10.56	0.402	1.61	8.81
		Acqua Panna	106	32.2	6.5	0.8	6.6	22
		Evian	360	79.9	26	1	6.62	12
		Tesla voda	604	130	53	4.7	45.8	90.5
	Sparkling	Heba Strong	3202.5	70	15.4	57.3	1060	199.8

		Radenska	2100	230	79	79	520	100
		Donat Mg	7500	390	1000		1500	2200
		Knjaz Miloš	1251	110	54	20.2	255	11
		K plus voda	400.8	66.2	39.5	1.01	4.56	20.4
		Aqua Gala	396.5	62.8	31.5	2	14.1	8.1
		Saint John's	377.2	78.1	21	1.28	11.4	7.5
		Jazak	423	80	41.8	3.8	6.1	24.7
		Karađorđe	1214	109	68	47.3	200.7	39
		Voda Vrnjci	959.8	67.84	51.32	30	200.6	39.96
		Jamnica	2246	114	43	27.1	805	116.1
		Sarajevski kiseljak	1805.6	248.5	41.3	16.8	598	490
		Mg mivela	2064.8	22.1	343	9.5	131.9	0.3
**Spain**								
	Still	Agua de Cuevas	246.9	47	25.4		1.6	13.5
		Agua del Rosal	295	65.4	13.7		45.2	
		Agua del Valle del Cardo		81	35		7	19
		Agua Sana	2.1	0.4	0.7		5.8	1.5
		Aguas de San Joaquín	165	35.7	16.5		8.3	3.7
		Aigua mineral Bonpreu	147.2	47.07	7.27		8.4	29.1
		Alzola	188	58.7	6.3		45.5	24.4
		AquaBona Fontoira	152	3.4	7.5		11	8.9
		AquaBona Fuenmayor	305	77	20.8		1.6	22.8
		AquaBona Peña Umbría	298	55	28		5.4	7.3
		AquaBona Santolin	274	92.4	2.73		2	6.5
		Aquadeus	323	74.2	23.4	0.9	3.3	20.4
		Aquarel	14.7	1.9	2.1		4.7	2.9
		Auchan	168	28.8	19.7	1.7	8	13.5
		Benassal	264	89.9	2.96		2.6	21.3

Bezoya	< 5	2.86	0.36		2.04	
Cabreiroà	193	10	5	2.1	58.2	
Caldes de Boì	34.6	5.4	0.5		21.7	13.2
Carrefour	266	21.5		3.5	94.7	11.5
Castrovita	118	39	2	< 1	2	3
Fonsana	38	7			7	3
Font Agudes del Montseny	253	58.9	16.8		29.7	41.2
Font de Pla Nova	270	74	36	1.2	11	
Font del Pi	317	67	66		27	207
Font del Regàs	129.2	32.9	4.1		13.5	10.2
Font d'Or	78.8	26.4	3.2		9.2	13.7
Font Jaraba	297	99.6	32		37.1	144
Font Major	173	54.5	7.7		9.5	14.2
Font Natura	205.6	70.9	15.7		8.9	57
Font Selva	249	34	6.8	1	54.9	14.6
Font Vella	167	43.2	11.5		1.3	
Fontecabras	295	90.1	37.5		32.6	117
Fontecelta	266	21.5		3.5	94.7	11.5
Fuensanta	162	58	4.5	0.9	5.8	17.5
Fuente Bruma	75.9	10.7	8.3		13.1	3.7
Fuente Liviana	268.3	65.4	17	0.4	0.8	19.2
Fuente Primavera	297.2	88.7	23.4		18.6	43.9
Fuentedueñas	246		7.4		3	6.7
Insalus	149	162	20.6		10.7	374
La Platina	79	16	16		9	18
Lanjaròn	108	28.9	11.4		5.9	
Les Creus	119	28	7.3	1	11.7	12.3
Lunares	292	96.3	35.7	2.3	39	142

		Marquesado	273.3	75.3	19.3	0.5	1.2	30.1
		Mondariz	156	7.7	5.5	5.1	49	1.7
		Monte Pinos	298	93.8	3.4		1.8	1.6
		Na Taconera	275	144	28		49	
		Ribagorza	331	67	27		24	18
		Ribes	146	46.6	7		5.6	
		San Vicente	109	14.9	9.8	2.6	14.2	9.9
		Sant Aniol	349	91.3	17.5		6.4	
		Sierra Cazorla	408.7	78.6	48.5	0.46	1.26	64
		Solan de Cabras	284	60	26.7	1	4.8	21.8
		Solares	249	75.2	15.6	1.7	91.1	37.2
		Sousas	77.7		1.1	1.8		
		Valtorre	174	22.1	22		32.5	22.3
		Veri	140	32.1	9.2		0.5	3.3
		Viladrau	113	27.7	4.5		11.9	11.2
	Sparkling	AquaBona Peña Umbría	298	55	28		5.4	7.3
		AquaBona Santolín	276	90.4	2.7		2.1	6.9
		Cortes de Arenoso	267.1	86.8	7.8	1	7.7	16
		Firgas	406	58.2	31.1		42.6	6.9
		Fonteide	53.6	6.5	4.1	9	21.3	4.9
		Fontenova	193.1	6.5	3.2	5.5	65	10.5
		Fonter	127	32	7.3		7.4	8.9
		Malavella	2214	53.7		49.4	1115	47.7
		San Narciso	2132	51.4			1080	47
		San Pellegrino	237.9	185.6	52.5	2.5	35	443.8
		Vichy Catalan	2081			50.7	1097	49.6
		Vilajuiga	1790	85.3	44.1	28	591	
**Switzerland**								

Still	Adelbodner Cristal	284	579	39	1.4	6.5	1268
	Adello Mineral	291	530	36.5	1.7	5.8	1127
	Allegra	344.2	100.4	23.7	0.7	2.6	59.3
	Appenzell Mineral Leise	407	108	17	0.7	3	3.8
	Aproz Cristal	250	360	70	2.5	6	930
	Aquella Cristal	245	300	75	2.5	4	840
	Valais Naturelle	225	90	22	2.5	7	110
	Aquina Rot	246	174	33.5	2.8	4.4	362
	Arkina Blau	355	85	25.7	0.8	8.9	40.8
	Cristallo Still	254	221	65.4	2.7	4.3	597
	Cristalp without CO2	227.6	115.3	40.7	1.9	21.9	240.7
	Eden	91.8	33.5	3.1	2.9	4.3	29.5
	Elmer Mineral without CO2	242	118	6.6	0.6	2.5	117
	Eptinger Still	278	510	117	2.5	4.2	1445
	Farmer Mineral Blau	287	546	35		6	1256
	Henniez Blau	389	104	20	1	7	12
	Knutwiler Grün	375	89	24	1.2	5	20
	M-Budget without CO2	80	100	20	0.5	5	280
	OK.- Mineralwasser Blau	194	37.3	15.2	0.98	4.42	8.7
	Saguaro Nature	277	136	42		1.8	268
	San Clemente Naturale		8.4	1.5		1.8	7.8
	Swiss Alpina Rot	239	208	37	1.9	5.2	480
	Valser Silence	155	53.7	1.5	0.2	0.2	10
	Zurzacher Naturelle	278	53.3	7.8	5.8	154	170
Sparkling	Adelbodner Mineral	284	579	39	1.4	6.5	1268
	Adello Mineral	291	530	36.5	1.7	5.8	1127
	Allegra Finin	344.2	100.4	23.7	0.7	2.6	59.3
	Appenzell Mineral Laut	407	108	17	0.7	3	3.8

	Aproz Classic	250	360	70	2.5	6	930
	Aquella Classic	245	300	75	2.5	4	840
	Aquina Blau	246	174	33.5	2.8	4.4	362
	Arkina Grün	355	85	25.7	0.8	8.9	40.8
	Cristallo Prickelnd	254	221	65.4	2.7	4.3	597
	Cristella Blau	249	287	71			798
	Cristalp with CO2	227.6	115.3	40.7	1.9	21.9	240.7
	Elmer Mineral with CO2	242	118	6.6	0.6	2.5	117
	Eptinger Prickelnd	278	510	117	2.5	4.2	1445
	Farmer Mineral Rot	287	546	35		6	1256
	Henniez Rot	389	104	20	1	7	12
	Knutwiler Blau	375	89	24	1.2	5	20
	M-Budget with CO2	235	95	26	2	7	140
	Meltinger	0.4	581.61	91	2	4.95	1443
	OK.- Mineralwasser Rot	194	37.3	15.2	0.98	4.42	8.7
	Passugger	771.3	210.8	22.4	2.7	41.2	50.7
	Prix Garantie	263	257	44.5	1.9	3.9	573
	Rhäzünser	1196	230	48.6	7.1	153	141
	Saguaro Classic	277	136	42		1.8	268
	San Clemente Frizzante		8.4	1.5		1.8	7.8
	Swiss Alpina Blau	239	208	37	1.9	5.2	480
	Valais Pétillante	225	90	22	2.5	7	110
	Valser	366	435	54.5	1.4	9.4	988
	Zurzacher Classic	270	12.4	0.2	6.3	282	258
**Turkey**							
Still	Ice Mountain	150	40.8	7.5	1.7	17.8	6
	Bleu	134	29		2	4	3
	Cactus	112	28	1.9	2.2	2.8	3

	Dassani	111	32	2.1	1.8	0	2.8
	Meadows	289	59	24	0.4		7
	Evian	71	11.5	8	6.2	1.15	8.1
	Volvic	125	33	2.1	2.3	2.6	2.3
	Cano	195	37.1	15.6	2.7	4.5	9.9
	Fiji	15.2	1.8	1.5	0.5	1.8	0.1
	Aqua	64	15	5.7	2.1	12	
	pH Balancer		1.5	1.2	1.9	1.2	
	pH Infinity	71	17.5	3.2	5.2	17.5	9.1
	Vitel	384	240	42	2.2	5.2	400
	Life	125	32	2.1	2.3	2.8	3
Sparkling	H-two-O	45	5		45	128	
	Cano	205	37.1	15.6		4.5	
	Oldenladia					123	
	Gerolsteiner	1816	348	108	11	118	38
	Evian Blue	360	80	26	1	6.5	14
	Badoit	1250	153	80	11	189	35
**United Kingdom**							
Still	Aqua Pura	20	14	3.5	2.5	12	14
	Aquavia	74.25	1.4		0.36	65	
	Buxton	248	55	19	1	24	13
	Evian	360	80	26	1	6.5	14
	Harrogate	215	57	19		8	13
	Highland	150	40.5	10.1	0.7	5.6	5.3
	Hildon		98.5	1.62		7.03	6.6
	Nestlé PureLife	184.6	59	10	1.2	11.9	9.7
	Sainsbury's Caledonian	240	55	16	2	15	28
	Tesco Ashbeck	25	11	3.5	2.5	10	11

	Volvic	74	12	8	6	12	9
Sparkling	Buxton	248	55	19	1	24	13
	Harrogate	215	57	19		8	13
	Highland Spring	150	40.5	10.1	0.7	5.6	5.3
	Hildon Natural		104	1.8		7.47	9.01
	Sainsbury's Caledonian	240	55	16	2	15	28
	San Pellegrino	245	174	51.4	2.2	33.3	430
	**AUSTRALA**						
**Australia**							
Still	K2 sport life	130	24	3.3	2.2	41	8.1
	Acqua Panna	106	32	6.5	0.8	6.6	22
	Islantic Water		6.4	2.4	0.6	11	3.4
	Pureau	0				0	
	Aqua love					12	
	Alka Power	1	7.5	0.05	0.2	1	
	Balance						
	Evian	360	80	26	1	6.6	14
	Voss	12	4	1	0	4	5
	Fiji	152	18	15			
	Yaru		39	18			
	Tasmanian Mountain Water	31.92	4.6		3.79		
	Moores ultra pure		0	0		0	
	Just Water Spring	140	17	25	9	29	
	Ossa spring		31.95			3.79	
	E'stel	90	2.2	0.03	0.13	42	1.3
Sparkling	Nu					1.9	
	Capi Mineral	200	20	29	21	31	6
	Perrier	420	150	3.9	1	9.6	25.3

		Mount Franklin					5	
		Santa Vittoria	185	36.5	18.5	0.45	0.61	
		Sparkling Italian	226	41.2	22.3	0.2	0.95	
		Voss	240	4	1	0	9	5
		San Pelligrino	243	164	49.5	2.2	31.2	40
		Yarra Sparkling		39	18			
		Coles carbonated mineral				10		
		Ossa sparkling		31.95			3.79	
**NORTH AMERICA**								
**Canada**								
	Still	Gerolsteiner	1816	348	108	11	118	38
		San Benedetto	296	51	29	0.97	6	4.2
		Glace			0.2		1.02	0.85
		Saint-Justin	560	7	6	3	415	0
		Galvanina	330	82	23	1.3	15	28
		Jackson Springs	210	42	13.2	1.3	0	0
		Zagori	244	72.8	3.8	0.62	1.96	6.33
		Eska	82	25	4	1	3	8
		Thunderbird	30	11	0.4	0.1	1	2
		Icelandic glacial	33	6	2	1	11	3
		Aquadeco	170	48	19	1.6	3.6	14
		Fuji	152	18	15	5	18	1
		Flow water	298	73	29	2	8	11.1
		waiakea	34	6	3	2	6	4
		De l'Aubier	35	2	2	16	5	2
	Sparkling	Gize	115	286	9.5	1.9	36.1	692
		Perrier	420	150	4	1	10	25
		San Pellegrino	243	164	49.5	2.2	31.2	402

	Nestlé PureLife	2	13	4.4	0	6.5	42
	Abenakis	70	90	60	15	720	125
	Zagori	237	86.1	5.9	0.7	2.1	7.8
	San Benedetto	296	51	29	0.97	6	4.2
	San Giorgio	176.6	27.8	26	2.5	52.8	11.4
	Penguin Ice	107	48	22	3.8	18	30
	Clearly Canadian	190	42	16	4.2	4.9	18
	Montellier	77	42	12	1	10	17
**United States**							
Still	Dasani						10
	Poland Spring		5.6	0.84		5.6	5
	Acqua Panna		32	6.4		6.9	21
	Arrowhead	81.1	20.4		1.5	11.1	3.8
	Deer Park		11	2.1		7.6	5.7
	Ice Mountain		11	2.1		7.6	5.6
	Nestlé PureLife		11	4.4		3.9	16
	Poland Spring Origin		4.8	1.3		1.6	3.8
	Fiji	155	18	15	4.9	18	0.7
	Zephyr Hills						
	Mountain Valley	230	68	7.4	1.3		9
	Crystal Geyser	69		5.7	1.5	12	2.1
	Hawaiian Springs		6.4	3.4	2.3	6.7	4.6
	Evian	360	93	32		6.7	13
	Volvic	76	13	8.7	6.5	12	8.8
Sparkling	Perrier		140	3.1		8.3	24
	Poland Spring		6.9	0.99			5
	San Pellegrino		170	45	2.4	31	420
	Zephyr Hills						1.8

	Arrowhead		37	5.6	2.1	11	11
	Deer Park		6.4	2.4		3.3	20
	Ice Mountain		6.4	2.4		3.3	20
	Nestlé PureLife		14.9	4.29			25.5
	Topo Chico		120	11	3.6		180
**SOUTH AMERICA**							
**Brazil**							
Still	Crystal	105.81	4.72	0.94	2.3	34.4	1.56
	Nestlé Pureza Vital	169.14	31	16.5	3.99	4.18	1.11
	Minalba	110.78	16.8	11.1	1.24	1.21	0.13
	Prata	50.71	8.5	4.07	2.76	2.39	2.95
	Bioleve	87.42	13.3	8.06	1.4	3.57	
	Ibira	79.81	6.02		4.4	91	
	Schin	52	8.99	1.29	1.42	6.80	1.31
	Inga	11.62	0.91	1.55	0.34	0.39	0.13
	Agua de Pedra	131.99	26.48	5.14	1.04	22.84	
	Agua Cristalina	1.98		0.05			0.93
	Vitalis	5.1	0.8	0.5		6.2	
	Aquarel	13.2	2.2	2.3		4.7	3.7
	Fonteide	56.9	5.8	4.3	8.5	20.3	4.9
	Serra Catarinense	95.49	18.68	4.71	0.57	6.77	0.36
	Raposo	21.62	3.43	2.10	1.22	3.34	4.35
	Serra da Estrela	16.5	2.7			4.4	
	Leve-Pura da Natureza	36.39	5.75	2.42	4.78	5.2	
	Fonte Pouso Alto	34.19	4.40	3.10	1.36	3.33	2.35
	Petropolis	7.14	3.85	2.20	2.50	13.93	1.82
Sparkling	Crystal	100.53	4.17	0.999	2.48	16.09	1.75
	Minalba	105.15	17.14	9.83	1.3	1.1	0.2

		Prata	91.29	15.3	7.15	3.78	7.61	6.88
		Bonaleve	10.23	2.08	0.542	0.845	1	
		Sao Lourence	283.73	28.17	12491	31.35	36.04	2.17
		1x Soda		13.9	3.9	3.1	26.1	38.12
		Agua de Pedra	122.83	25.18	4.44	1.09	23.02	
		Perrier	445	155	6.8	1.3	11.8	46.1
		Raposo	91.52	26.04	2.87	1.47	3.94	6.14
		**ASIA**						
**India**								
	Still	Komin	197	36	22	0.1	1	3
		Kelzai Volcanic Water	70	29	1		32	1
		Mulshi		15.6	6.2	4	6.9	3.2
		Aava		17	11.5		50	
		Aion	58					
		Bisleri	170	13.6	7.8			19.3
		Aquafina		33.6	11.7	0.5	1.8	6
		Atlas Premium	466.6	16.6	7.8	2.6		
		Dew Drops	196.6	58.8	44.4	22.2	24.4	10.6
		Golden Eagle	50	22.2	37.7	2	1.3	34.4
		Golden Valley		8	4	4	8	4
		Maqua 2000	+	2				35.5
		Kinley			+		+	+
		Himalayan	33					33
	Sparkling	Perrier	390	147.3	3.4	0.6	9	
		San Pellegrino	243	164	49.5	2.2	31.2	
		Zoik		41.3	6.2	13		
		Boca	155	22	10		20	
**Saudi Arabia**								

Still	Highland Spring	150	40.5	10.1	0.7	5.6	5.3
	Speyside Glenlivet	0	12	1.6	0.7	3.9	4
	Nova	26	11	3.4	1.2	17	26
	Berain	50	22	3	5	17	9
	Arwa	7.7	<1.0	21.1	1	3	74.5
	Hada	30	13	4	0.8	20	20
	Nestlé	42	36	4.7	0.2	16.8	22
	Hana	18	21	9	8	5	28
	Tannourine	160	50	13	1	4	4
	Solan de Cabras	0	60	26.7	1	4.8	21.8
	Harrogate	211	45	14	0	29	32
	Naqi	25	14	6	1.6	2	31
	Voss	0	3.7	0.9	0	3.8	2.1
	Panda	25	6	2	1	29	30
	View	6.1	29.6	0.75	1.4	1	3
Sparkling	Highland Spring	150	40.5	10.1	0.7	5.6	5.3
	Souroti	744	182	54	11	78	
	Perrier	420	150	3.9	1	9.6	25.3
	San Pellegrino	0	166	49.5	2.1	30	401
	Montana	48	10	3.8	0.26	0.3	1.6
	Voss	0	3.7	0.9	0	122	2.1
	Berain	50	22	3	5	17	9
	Badoit	1250	153	80	11	180	35
	Aqua	0	296	86.8	5.16	9.62	34
	Kiseljak	1878.8	232.5	46.2	19.8	609	580
**Singapore**							
Still	Ice mountain	150	40.8	7.5	1.7	17.8	6
	Bleu	134	29		2	4	<3

	Cactus	112	28	1.9	2.2	2.8	<3
	Dassani	111	32	2.1	1.8	0	<2.8
	Meadows	289	59	24	0.4		7
	Evian	71	11.5	8	6.2	1.15	8.1
	Volvic	125	33	2.1	2.3	2.6	<2.3
	Cano	195.5	37.13	15.64	2.7	4.5	9.9
	Fiji	15.2	1.8	1.5	0.5	1.8	0.1
	Aqua	64	15	5.7	2.1	12	
	pH Balancer		1.5	1.2	1.9	1.2	
	pH Infinity	71	17.5	3.2	5.2	17.5	9.1
	Vitel	384	240	42	2.2	5.2	400
	Life	125	32	2.1	2.3	2.8	<3
Sparkling	Pocar sweat		20.04	6.07		43	1008.63
	Ion water		20.04	33	195.5	168	
	H-two-O	45	5		45	128	
	Cano	205	37.13	15.64		4.5	
	Oldenladia					123	
	Gerolsteiner	1816	348	108	11	118	38
	Evian Blue	360	80	26	1	6.5	14
	Badoit	1250	153	80	11	189	35
